# Acknowledgment to Reviewers of *Biomimetics* in 2020

**DOI:** 10.3390/biomimetics6010011

**Published:** 2021-01-31

**Authors:** Biomimetics Editorial Office

**Affiliations:** MDPI AG, St. Alban-Anlage 66, 4052 Basel, Switzerland

Peer review is the driving force of journal development, and reviewers are gatekeepers who ensure that *Biomimetics* maintains its standards for the high quality of its published papers. Thanks to the cooperation of our reviewers, in 2020, the median time to first decision was 21.5 days and the median time to publication was 53.5 days. The editors would like to express their sincere gratitude to the following reviewers for their precious time and dedication, regardless of whether the papers were finally published:
Abella, Laura SanchezCostantino, Marco FabioAdnan, MemicCui, HaitaoAhmadi, Seyed F.D’Aurelio, RobertaAlfredo, Gonzalez-PerezDahms, Hans-UweAli, JamelD’Angelo, IvanaAl-Obaidi, KaramDavid, BierbachAl-Radhi, Mohammed SalahDavidova, MarieAlwattar, Tahseen A.Degli Esposti, LorenzoAnne-Claire, GrooDen Toonder, JaapAntoniadis, AristomenisDeschner, JamesAstruc-Diaz, FannyDing, Xi-LunAtrian-Blasco, ElenaDiogo, PatríciaAttik, Nina G.Do, Thanh N.Babaei, MahnoushDong, GuoyingBae, HaejinDong, MingdongBalayeva, Narmina O.Ehlicke, FranziskaBarath, ZoltanEne, RazvanBarbieri, FilippoEstevez, Alberto T.Barrientos, AntonioFarzaneh, Helena H.Bastrzyk, AnnaFerreira, BarbaraBerthold-Pluta, AnnaFraga-García, PaulaBertl, AdamFrancolini, IolandaBhounsule, Pranav A.Fu, ChaoBohinc, KlemenFuest, MatthiasBonilla, Clara Eugenia PlazasGadomska-Gajadhur, AgnieszkaBonser, RichardGangadharan, RajanBottari, FabioGao, YunCampisi, GiuseppinaGebeshuber, IlleCanfield, StephenGhidelli, MatteoCarmagnola, IreneGhodake, GajananCataldi, AmeliaGiberti, HermesCerbu, CameliaGomes, Ana P.Chowdhury, Md RaihanGradov, Oleg V.Chu, Chun HungHasling, Karen M.Clegg, John R.Hayes, SamanthaCopot, CosminHerbig, MaikHesselberg, ThomasPatane, LucaHo, Chia-chePatnaik, Sourav S.Horner, AndreasPerez-Ramirez, CarlosHosseini Nejad, ElhamPitucha, MonikaIconaru, Simona L.Prado Da Fonseca, ViniciusJain, AnujQian, Jin-yuanJeronimidis, GeorgeRamachandran, RoshiniJi, Ai-hongRamos, LaurenceKarcz, DariuszReal Oliveira, Maria E. C.D.Khassawna, Thaqif E.Rey, JuanKhetani, Salman R.Robert, NewcombKim, Young-RokRobertson, JamesKohsaka, RyoRovalo, ErinKorupalli, ChiranjeeviRusu, Laura CristinaKrater, MartinSaeki, DaisukeKrieger, AxelSamal, SangramKucinska, MalgorzataSamuel, ShaniKumar, HarishSantarelli, AndreaLamas, María IsabelScribante, AndreaLauder, GeorgeShach-Pinsly, DalitLee, Jong YongShin, SangJoonLee, JunsooSimanjuntak, Firman MangasaLeech, DamienSingh, AnirudhaLiu, YuxinSkaba, DariuszLopez-Cara, Luisa CarlotaSlegers, Nathan J.Lord, Nathan P.Sodergard, AndersMagdysyuk, Oxana V.Sotomayor, MariaMagyari, KlaraSuarato, GiuliaManini, PaolaSwaileh, WassimMansoori, AliTampieri, AnnaMarciello, MarziaTanase, ConstantinMartin-Gomez, CesarTartaglia, GianlucaMayama, HiroyukiTawil, Bill J.Mazza, EdoardoTenkumo, TaichiMcHenry, MatthewThakar, DhruvMead, TarynThakur, VijayMishra, AlaknandaThiria, BenjaminMiyaji, AkimitsuToby, BrianMlynarczyk, DariuszTouchie, MarianneMondal, SayanTouloupaki, EleftheriaMonteiro, CarlosTsuji, HidetoMueller, JochenTursi, AntonioMuntean, AlexandrinaTytell, EricNakielski, PawelVan De Kamp, ThomasNaumowicz, MonikaVanaga, RutaNavabpour, ‪ParniaVerstegen, Monique M. A.Niclos Gutierrez, JuanVikraman, DhanasekaranNiewiarowski, PeterVisser, ArnoudNoskov, Boris A.Vladkova, TodorkaNurunnabi, MohammadWandelt, KlausOhya, YoshikazuWang, BingPastor, JESUSWeissburg, MarcWesthauser, FabianYuan, HaoboWie, Jeong JaeZhang, Sung-UkWilliams, JohnZhao, JieWu, Chia-WenZhu, HanxingYilmaz, MehmetZubareva, Ekaterina V.Yu, LuZwart, Hub

